# Tetanus in Southern Vietnam: Current Situation

**DOI:** 10.4269/ajtmh.16-0470

**Published:** 2017-01-11

**Authors:** Duong Bich Thuy, James I. Campbell, Tran Tan Thanh, Cao Thu Thuy, Huynh Thi Loan, Nguyen Van Hao, Yen Lam Minh, Le Van Tan, Maciej F. Boni, C. Louise Thwaites

**Affiliations:** 1 Hospital for Tropical Diseases, Ho Chi Minh City, Vietnam.; 2 Oxford University Clinical Research Unit, Ho Chi Minh City, Vietnam.; 3 Centre for Tropical Medicine and Global Health, University of Oxford, Oxford, United Kingdom.

## Abstract

In Vietnam, there are no accurate data on tetanus incidence to allow assessment of disease burden or vaccination program efficacy. We analyzed age structure of 786 tetanus cases admitted to a tertiary referral center in Vietnam for three separate years during an 18-year period to examine the impact of tetanus prevention programs, namely the Expanded Program on Immunization (EPI) and the Maternal and Neonatal Tetanus (MNT) initiative. Most cases were born before the initiation of EPI. Median age increased from 33 (interquartile range: 20–52) in 1994, to 46 (32–63) in 2012 (*P* < 0.001). Birth-year distribution was unchanged, indicating the same birth cohorts presented with tetanus in 1994, 2003, and 2012. Enzyme-linked immunosorbent assay measurements in 90 men and 90 women covered by MNT but not EPI showed 73.3% (95% confidence interval [CI]: 62.9–82.1%) of women had anti-tetanus antibody compared with 24.4% (95% CI: 15.9–34.7%) of men, indicating continued tetanus vulnerability in older men in Vietnam.

Tetanus is a vaccine-preventable disease occurring rarely in high-income settings but is a continuing concern in many low- and middle-income countries (LMICs).^1^ Except for neonatal tetanus, where there is a well-established surveillance system, the true global incidence of tetanus is unknown. In Vietnam, prevention of tetanus relies on two main programs: the Expanded Program on Immunization (EPI), and the Maternal and Neonatal Tetanus (MNT) elimination initiative. The EPI was introduced to Vietnam in 1981,^2^ and aims to protect children using a primary vaccination series given at 2, 3, and 4 months of age. However, subsequent boosting is necessary for long-term immunity.^3^ In Vietnam, consistently high rates of primary EPI vaccination coverage (DTP3) have been reported,^2^ but no data are collected regarding uptake of any subsequent boosters. Evidence from other LMIC settings suggests that without boosting approximately 50% of the population will have subprotective antibody levels by 6–15 years of age.^3^,^4^ The MNT elimination initiative aims to prevent tetanus in women and neonates by vaccination of pregnant women or women of child-bearing age.^5^,^6^ The initiative has achieved remarkable success throughout the world. Vietnam achieved World Health Organization (WHO) Maternal and Neonatal Elimination Status in 2005, although the disease has not been completely eradicated and occasional cases of neonatal tetanus continue to be reported.^1^,^7^–^9^

In Vietnam, implementation of the MNT elimination initiative involved improving surveillance systems for neonatal tetanus. However, there are no accurate incidence data on non-neonatal tetanus, and the efficacy of Vietnam's tetanus prevention program is unknown. Outbreaks of notifiable diseases such as measles serve as proxy indicators, but these are affected by herd immunity effects, which are not applicable to tetanus. A recent health economic analysis of the benefits of the EPI program in Vietnam excluded tetanus from analyses as the effects of the EPI were difficult to distinguish from those of the MNT initiative.^10^

The Hospital for Tropical Diseases Ho Chi Minh City (HTD) is a tertiary referral hospital and receives tetanus cases from the whole of southern Vietnam. Over recent years, admissions of tetanus have remained high, with over 200 cases annually; similar to figures reported 20 years ago.^11^ In view of the lack of accurate published data on incidence of tetanus in LMICs and to estimate the impact of the EPI and MNT programs in Vietnam, we carried out a study examining patient demographics over an 18-year period and measured anti-tetanus antibodies in a population sample old enough to have been born prior to the institution of the EPI program but young enough to have reached child-bearing age after the initiation of MNT initiative in 1991.

This study was approved by the Scientific and Ethics Committee of HTD. Demographic data for all cases of tetanus admitted to HTD between January 1 and December 31 for years 1994, 2003, and 2012 were collected. Although accurate data collection began in 1993,^11^ data were only available for 9 months in that year and to have a complete year for analysis, 1994 was chosen. Recently in 2012 complete data were available and the year 2003 was selected as it was the midpoint between 1994 and 2012. Data from years 1994 and 2003 were collected prospectively onto special case record forms and stored in an electronic database. Data for 2012 were retrieved from the hospital database. No data were available on neonatal patients in 1994, therefore, cases occurring in infants < 1 year old were excluded from all years. Both data sources distinguished between patients discharged alive and those discharged home against medical advice and felt likely to die by attending staff. These latter events were classified as “deaths.”

To ascertain the degree of tetanus protection in the population most at risk of tetanus, anti-tetanus antibodies were measured in a random sample of 90 men and 90 women born between 30 and 45 years (birth years 1984 or older) using specimens from long-term general population serum collection that has been ongoing in Ho Chi Minh City since 2009.^12^ Although EPI was initiated in Vietnam in 1981, coverage in 1984 was still only 5%.^2^,^13^ Thus, individuals in this cohort were selected as they were unlikely to be vaccinated under the EPI program, but should have been covered by the MNT initiative. Antibodies were measured using nonquantitative enzyme-linked immunosorbent assay (ELISA) as described previously.^14^

Records were retrieved from a total of 790 patients with tetanus ≥ 1 year old. Four cases from the year 2003 were excluded due to insufficient data, leaving a total of 786 cases for analysis. Mortality rates fell during the study from 91/322(28.3%) in 1994, to 20/245 (8.2%) in 2003 and 17/219 (7.8%) in 2012. This coincided increased availability of mechanical ventilation between 2000 and 2002.^11^

Male:female ratios were 1:2.12, 1:2.22, and 1:3.4 for the years 1994, 2003, and 2012, respectively. Median (interquartile range [IQR]) age of patients increased over the study period from 33 (20–52) in 1994, to 35 (22–54) in 2003 and 46 (32–63) in 2012 (*P* < 0.001; one-way analysis of variance [ANOVA]). When the 2012 admissions were analyzed according to gender, a particularly marked reduction was noted in women born before 1981: 5/50 (10%) admissions were female in 2012 compared with 15/76 (19.7%) in 2003 and 22/103 (21.3%) in 1994.

The distribution of birth year for patient admissions is shown in Figure 1

**Figure 1.**
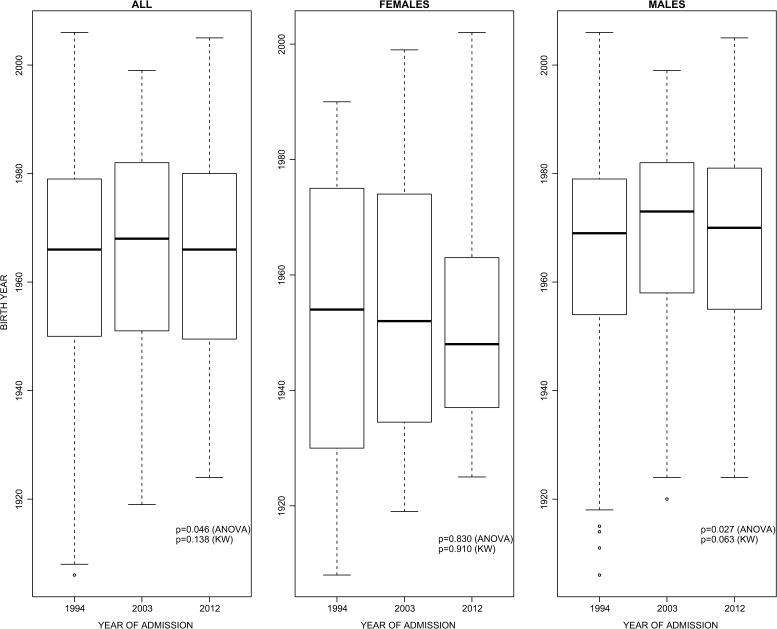
Birth year of tetanus admissions (≥ 1 year of age) admitted to the Hospital for Tropical Diseases by year. Effect of admission year on birth year was tested with a one-way analysis of variance (ANOVA) and a Kruskal–Wallis (KW) test. The birth cohorts that represent tetanus admissions do not appear to be changing over an 18-year period.

. The majority of cases (75%) were born before 1981. After correction for multiple comparisons, there was no statistical support for the birth year distribution changing by year of admission (one-way ANOVA, Kruskal–Wallis test). Even if a larger sample size had made these associations significant, the median birth year (1967, across all patients) did not change by more than 1 year when stratifying the data by year of admission. Median birth year for males was 1970 and for females was 1952. In other words, the same birth cohorts (mainly, pre-EPI cohorts) were being admitted for tetanus infection over an 18-year period despite the entire population aging by 18 years during this time.

ELISA results are shown in Figure 2

**Figure 2.**
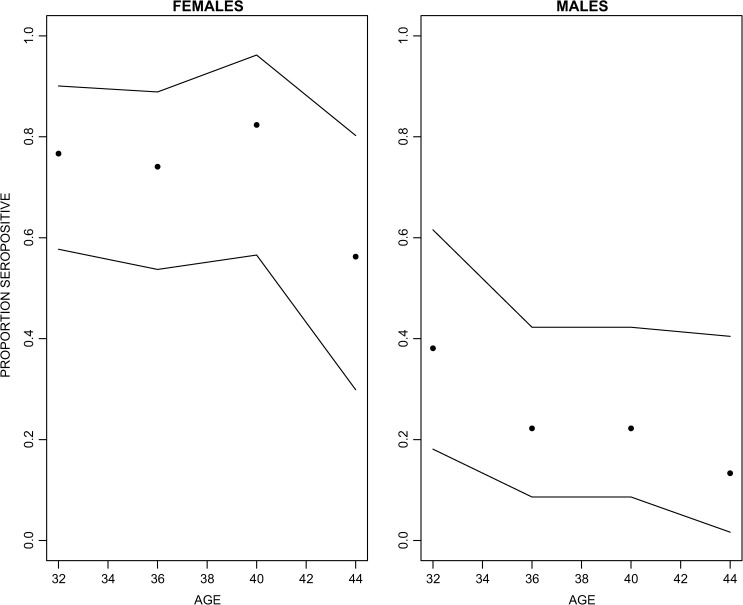
Anti-tetanus antibody protection in 90 males and 90 females from a bank of general-population serum samples for Ho Chi Minh City. Individuals are grouped into 4-year age bands. Confidence intervals are plotted with the exact binomial method.

. Of women born before 1985, 73.3% (95% confidence interval [CI]: 62.9–82.1%) had detectable anti-tetanus antibody compared with 24.4% (95% CI: 15.9–34.7%) of men.

We report that significant numbers of tetanus cases continue to occur in southern Vietnam. Although there are relatively robust methods of recording cases of neonatal tetanus, in many countries, the reporting of tetanus in other age groups remains inaccurate with significant underreporting.^1^,^15^ In 2012, the WHO reported a total of 186 cases in Vietnam as a whole, 33 cases fewer than admitted to our hospital alone.^16^ Improved surveillance has been a crucial element in the success of the MNT initiative and if extended to all tetanus cases, it would provide evidence of the need for wider vaccination programs, and enable more accurate evaluation of their success.^17^,^18^

Consistently high tetanus vaccination coverage has been reported in Vietnam (range approximately 90–99% since 1994 for EPI and 89–93% for MNT^2^), and our data suggest this appears to be having a significant impact. The majority of cases still occur in individuals born before 1981. Antibody measurements showed a large difference between men and women born before the EPI program achieved widespread coverage. In 2012, only 5/50 of female admissions (10%) were born after 1981 compared with 47/219 (21%) of male admissions suggesting there continues to be an extra benefit of the MNT initiative.

Our study is limited in that we were not able to ascertain the number of tetanus cases that did not present to our hospital and may have been treated in private facilities, provincial hospitals, or may not have attended health-care facilities. It is likely that, as facilities for care have improved over the 18-year study period, an increased proportion of tetanus cases are being cared for in other hospitals and we have underestimated the true regional incidence. We also only examined disease impact from a health-care perspective but the continuing occurrence of tetanus has other consequences. The median hospital cost of patients in our study was 521 U.S. dollars (IQR: 190–1,799) mostly paid directly by patient's relatives. When compared with a construction worker's average monthly salary of 215 U.S. dollars, this represents an additional financial and social burden.^19^

Nevertheless, although a significant number of cases of tetanus continue to occur in southern Vietnam, there is evidence of the efficacy of prevention programs, particularly in women. Improved understanding of how this is occurring and which individuals remain at risk would improve the design of vaccination catch-up programs and help reduce the burden of this entirely preventable disease.
